# Supplementary figures and images for: The Autonomous Parvovirus Minute Virus of Mice Localizes to Cellular Sites of DNA Damage Using ATR Signaling
Source: Viruses. 2023 May 25;15(6):1243. doi: 10.3390/v15061243 (PMC10300767; doi:10.3390/v15061243)

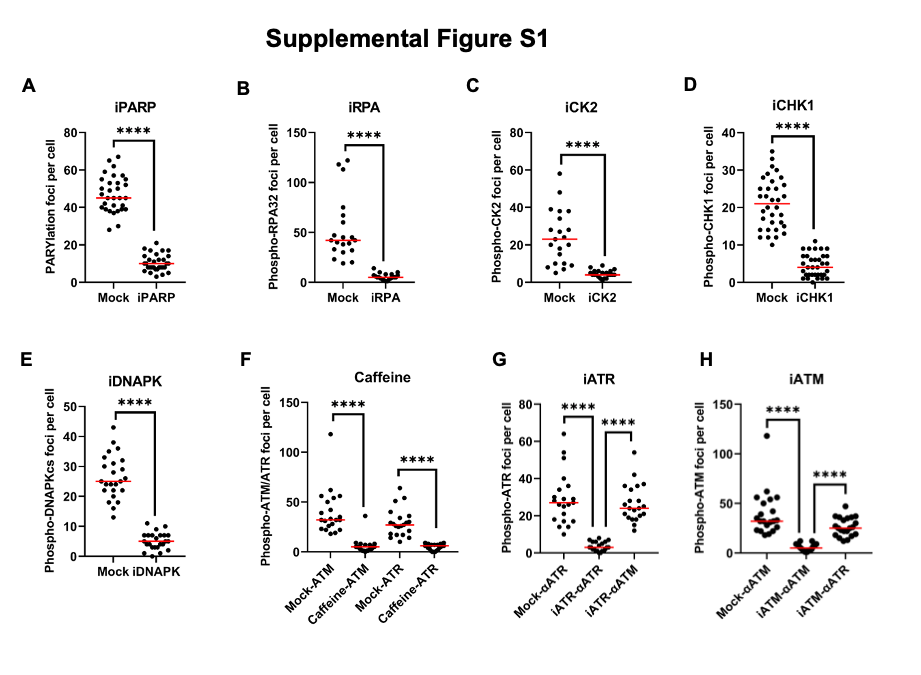

Supplement: Supplementary file 1 [file viruses-15-01243-s001.zip › Supplementary figures/Figure_S1.tiff]

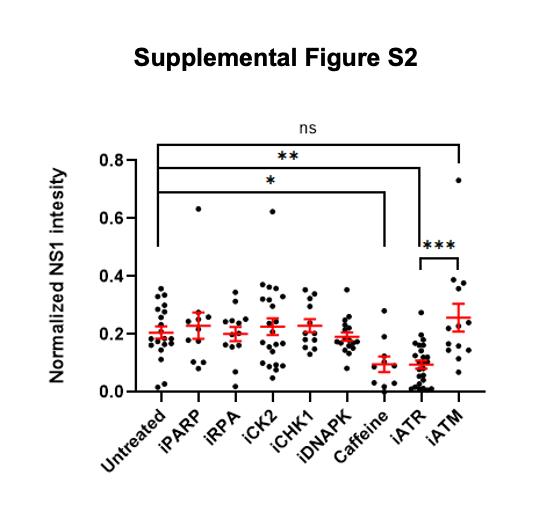

Supplement: Supplementary file 1 [file viruses-15-01243-s001.zip › Supplementary figures/Figure_S2.tiff]

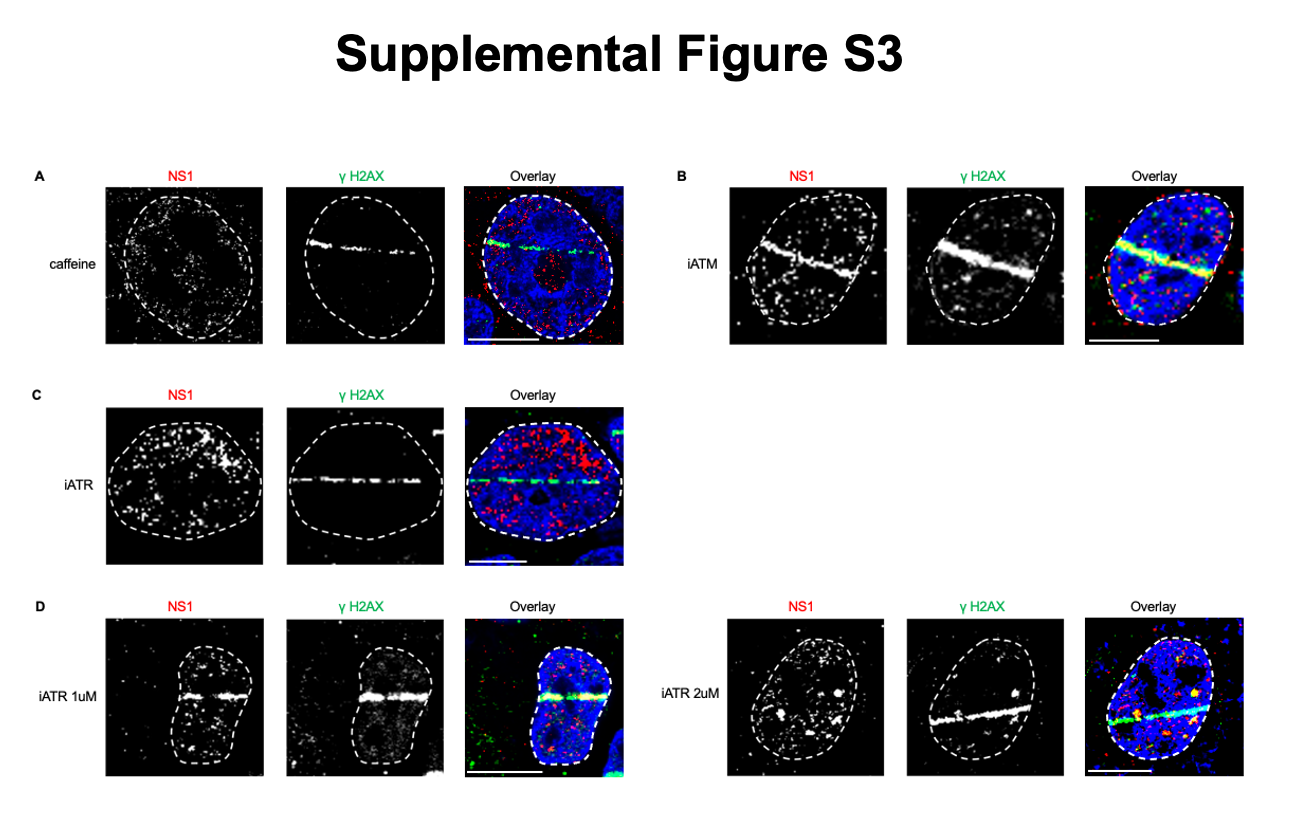

Supplement: Supplementary file 1 [file viruses-15-01243-s001.zip › Supplementary figures/Figure_S3.tiff]
